# Frequency, stability and differentiation of self-reported school fear and truancy in a community sample

**DOI:** 10.1186/1753-2000-2-17

**Published:** 2008-07-14

**Authors:** Hans-Christoph Steinhausen, Nora Müller, Christa Winkler Metzke

**Affiliations:** 1 Department of Child and Adolescent Psychiatry, University of Zurich, Neumuensterallee 9, CH 8032, Zurich, Switzerland

## Abstract

**Background:**

Surprisingly little is known about the frequency, stability, and correlates of school fear and truancy based on self-reported data of adolescents.

**Methods:**

Self-reported school fear and truancy were studied in a total of N = 834 subjects of the community-based Zurich Adolescent Psychology and Psychopathology Study (ZAPPS) at two times with an average age of thirteen and sixteen years. Group definitions were based on two behavioural items of the Youth Self-Report (YSR). Comparisons included a control group without indicators of school fear or truancy. The three groups were compared across questionnaires measuring emotional and behavioural problems, life-events, self-related cognitions, perceived parental behaviour, and perceived school environment.

**Results:**

The frequency of self-reported school fear decreased over time (6.9 vs. 3.6%) whereas there was an increase in truancy (5.0 vs. 18.4%). Subjects with school fear displayed a pattern of associated internalizing problems and truants were characterized by associated delinquent behaviour. Among other associated psychosocial features, the distress coming from the perceived school environment in students with school fear is most noteworthy.

**Conclusion:**

These findings from a community study show that school fear and truancy are frequent and display different developmental trajectories. Furthermore, previous results are corroborated which are based on smaller and selected clinical samples indicating that the two groups display distinct types of school-related behaviour.

## Introduction

School refusal is defined as difficulty attending school associated with emotional distress, especially anxiety and depression. This type of school absenteeism is observed in some 1 to 5 percent of children, predominantly in 5 to 6 and in 10 to 13 year olds, and tends to be more common in females [1-3]. Terms such as separation anxiety and school phobia have been used interchangeable with school refusal. In the present report, the term school fear will be used because self-reported school-related fear led to the definition of one of the groups under study.

Truancy generally refers to unexcused, illegal absence of school linked to lack of parental knowledge about the behaviour [3]. Accurate estimates of the prevalence of truancy are lacking due to inconsistent tracking and reporting practices of schools. A recent study from the US used self-reported data and found nearly 11% of 8^th ^graders and over 16% of 10^th ^graders reported truancy within the past 4 weeks [4]. School absenteeism is a broader term that includes both school refusal and truancy.

Given the limited knowledge about the correlates and/or causes of school fear and truancy, the present study used data from a large Swiss epidemiological survey that is predominantly based on self-reported data in young people. The Zurich Adolescent Psychology and Psychopathology Study (ZAPPS) is based on a theoretical model in order to study conditions and processes that are essential to the mental health of growing young people as well as to the development of mental problems and disorders. A broadband questionnaire was chosen in order to obtain information on relevant behavioural and emotional problems of adolescents. In order to analyze potential risk, compensatory, vulnerability, and protective factors of psychopathology [5]. life events were hypothetically seen as stressors, and various psychosocial variables including coping and self-related cognitions, and features of the social network including parents and school environment were regarded as moderating factors with regard to behavioural and emotional problems. This model and data set was used as a background for the present study aiming at a differentiation of school fear and truancy based on self-reported behavioural data.

Previous studies on school absenteeism have addressed some of the issues that are most relevant for the present study, namely, coexisting psychopathology, stressful life events, personality features, family characteristics, and features of the school environment. In terms of coexisting psychopathology there is a strong though not exclusive association of school refusal with internalizing disorders including anxiety disorders and depression [6-9] and an even stronger association between truancy and externalizing disorders including antisocial behaviour and substance abuse [10-12].

Very little is known about the relevance of stressful life events. According to Huffington and Sevitt [13] there is a tendency for critical life events to occur more frequently among truant pupils rather than school refusers. Furthermore, there is also only a paucity of studies dealing with personality features indicating that school absenteeism is linked to negative self esteem [14,15].

A few systematic studies dealing with family characteristics have found that absentee students frequently come from families with single mothers [11,12,16-18] and perceive less parental acceptance but more family conflicts [18]. Unclear role definitions and performances have been observed in the families of school refusers [16,19] and there is some evidence that truancy is associated with parenting deficiencies, delinquency and violence among the parents, and other indicators of familial disadvantage [15,20,21].

Finally, the effects of school environment on school refusal and truancy have not received much scientific attention. A restrictive school climate, marked competition among pupils, pronounced control and lack of support by the teacher have been notified to be associated with truancy and school absenteeism [22,23].

Given the small number of existing empirical data, the present study had the following aims: (1) the assessment of the frequency and stability of self-reported school fear and truancy across three years of time in a large community survey, and (2) the identification of differential features of these two forms of school absenteeism with respect to emotional and behavioural problems, stressful life events, personality features, perceived parenting behaviour, and qualities of the school environment.

## Method

### Subjects

Originally, the present sample is based on a cohort of 1,964 pupils aged 6 to 17 who were living in the Canton of Zurich, Switzerland in 1994. The cohort was a stratified randomized sample representing the 12 counties of the canton, the school grades, and the types of school and formed the basis of the Zurich Epidemiological Study of Child and Adolescent Psychopathology (ZESCAP). A full description of details of the sampling procedure was given in a previous article [24].

The preadolescents and adolescents (aged 11 – 17 years) of the ZESCAP sample (N = 1,110) provided the basic cohort of the longitudinal Zurich Adolescent Psychology and Psychopathology Study (ZAPPS). So far, this longitudinal study had assessments at time 1 in 1994, time 2 in 1997, time 3 in 2001, and time 4 in 2005. In the present study, data from the first two waves only were used because most of the subjects no longer were attending school at times 3 and 4.

From this cohort a total of N = 832 subjects participated at times 1 and 2 of the study. Mean ages were 13.6 (SD = 1.6) years at time 1 and 16.6 (SD = 1.6) years at time 2. This sample was composed of 403 (48.4%) males and 429 (51.6%) females. The missing sample at time 2 was composed of more males than females (37 vs. 28%) and older subjects (Mean age 14.3 vs. 13.6 years) whereas participants and non-participants did not differ significantly on the two defining items of school fear or truancy. These two items were derived from the Youth Self Report (YSR, see below). For school fear, a score of 2 on item 30 (I am afraid of going to school) was indicative whereas truancy was defined by a score of 2 on item 101 (I cut classes or skip school).

Based on these definitions, a total of N = 104 subjects showing some form of school absenteeism were found at time 1. This subsample was composed of N = 57 subjects with school fear, N = 41 subjects who were truant, and N = 6 subjects who fulfilled both criteria. Because of the very small sample size the latter group was not included in the analyses. At time 2 the total subsample of pupils showing some form of school absenteeism amounted to N = 200 with N = 30 fulfilling the criterion of school fear, N = 154 showing truant behaviour, and N = 16 showing both sorts of behaviour. Again, the latter group was not considered in the analyses. The resulting samples are described in Table 1. In addition to the four different groups showing school absenteeism at either time 1 or 2, two control groups at the two times of assessment were included in the study. Due to significant age and sex differences between the two groups with school absenteeism it was decided to place the control groups between the two former groups with regard to age at time 1 and sex.

**Table 1:** Sample characteristics

	Students with School Fear (1)	Students with Truancy (2)	Controls (3)	F	p	Post hoc tests
Time 1						
N	57	41	48			
Age (Mean ± SD)	13.2 ± 1.5	14.5 ± 1.4	13.6 ± 1.3	11.0	<.001	2>1,3
Sex (N)						
Males	21 (37%)	18 (44%)	16 (33%)			
Females	36 (63%)	23 (56%)	32 (67%)			
						
Time 2						
N	30	154	90			
Age (Mean ± SD)	16.3 ± 1.7	17.3 ± 1.6	17.0 ± 1.4	5.96	.003	2 >1
Sex (N)						
males	7 (23%)	62 (40%)	35 (39%)			
females	23 (77%)	92 (60%)	55 (61%)			

### Measures

The ZAPPS is based on a theoretical model in order to study those conditions and processes that are essential to both mental health and mental problems of growing young people. A broadband questionnaire was chosen in order to obtain information on relevant behavioural and emotional problems of adolescents. In order to analyze potential risk, compensatory, vulnerability, and protective factors of psychopathology [5], life events were hypothetically seen as stressors, and various psychosocial variables including coping, self-related cognitions, and features of the social network were regarded as moderating factors with regard to behavioural and emotional problems.

Questionnaires were filled out confidentially by the subjects during school hours at time 1 and had to be mailed at later waves of assessment. All questionnaires reflect raw scores and are positively keyed, i.e. high scores represent high expression of the content of the scale. Normative information is missing for all scales except the Youth Self – Report. Thus, it was decided to use raw scores for all analyses in the present study.

#### Youth Self – Report (YSR)

The problem behaviour section of the YSR [25] and its Swiss adaptation [26] consist of 112 items scored 0 (not true), 1 (somewhat or sometimes true) and 2 (very true or often true) reflecting the following primary subscales: socially withdrawn, somatic complaints, anxious/depressed, social problems, thought problems, attention problems, delinquent behaviour, and aggressive behaviour. Two second-order scales reflecting internalizing and externalizing can be calculated. Alpha coefficients of internal consistency ranged from 0.61 to 0.93 across scales and time.

#### Life Event Scale (LES)

A total of 36 items were chosen from pre-existing questionnaires on life events. The time frame was defined as the twelve months prior to filling out the questionnaire. Beside frequencies of life events, a total impact score was calculated. This was based on a scale attached to each item ranging from -2 to +2 and indicating how unpleasant or pleasant the respective event was [27]. The alpha coefficients for the total impact score ranged from 0.68 to 0.71 at the three times of the assessment.

#### Self – Related Cognitions (SRC)

The ten-item scale for the measurement of self-esteem by Rosenberg [28] and items from a German questionnaire assessing self-awareness [29] were further included into the questionnaire. The latter scale assesses introspective capacities for one's feelings, actions, and past. Alpha coefficients for the two scales across the three assessments ranged from 0.77 to 0.89.

#### Perceived Parental Behavior (PPB)

Based on pre-existing literature, we developed an inventory that consisted of 32 items [30]. Factor analysis resulted in 3 factors explaining 34% of the variance for mothers and 35% of the variance for the fathers. Alpha co-efficients of internal consistency ranged between 0.70 and 0.83. The 3 scales were labelled "acceptance" (e. g., "my mother/father praises me when I do something good"), "rejection" (e. g. "my mother/father easily becomes upset if I don't do what she/he says") and "control" (e. g. "my mother/father has clear rules for my behaviour").

#### Perceived School Environment (PSES)

These scales were derived from a German project on development in adolescence [31] and consist of 32 items that deal with the perceived psychosocial qualities of the school environment. Our own factorial analyses re-identified the 5 factors and the resulting scales had Alpha coefficients of between 0.64 and 0.78. The 5 scales are labelled "competition among pupils" (e. g. "in our class, each student tries to be more successful than the other"), "control by the teacher" (e. g. "many of our teachers treat us like small children"), "performance stress" (e. g. "we hardly manage our homework"), "possibility to participate " (e. g. "our teachers ask for our opinion before deciding"), and "peer acceptance" (e. g. "I consider myself to be one of the most accepted students in our class").

### Statistical analyses

Stability of school refusal and truancy across time was tested by McNemar tests. Group comparisons were performed by multivariate analyses of covariance (MANOVA). In a first step, age was tested as a covariate in order to control for within group differences. However, because of not affecting any group differences age was not considered anymore in the final analyses. Gender was considered as another covariate but resulted only in rather few and marginal interaction effects with group so that it will not be considered in the presentation of findings. All analyses are based on standardized T-scales (Mean = 50, SD = 10).

## Results

### Frequency and Stability of School Absenteeism

**Figure 1:**
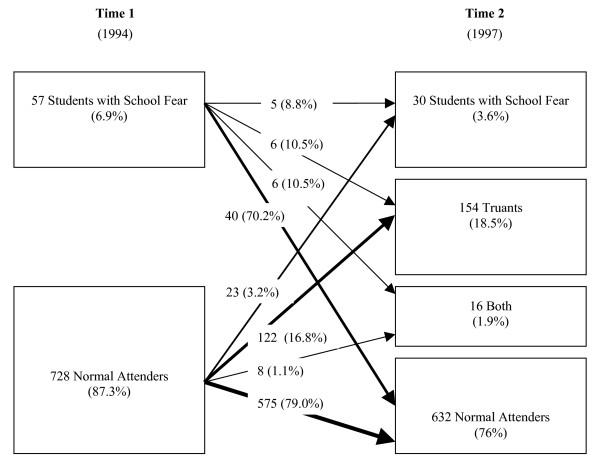
Stability of school fear from time 1 to time 2.

The frequency of school fear was 6.9% at time 1 and 3.6% at time 2. The stability of school fear is shown in figure 1. The number of subjects showing this behaviour was significantly decreasing from time 1 to time 2 (McNemar p < .001). At both times the number of females showing school fear was significantly increased (see Table 1, time 1 Chi^2 ^= 3.82, p < .05; time 2 Chi^2 ^= 9.52, p < .01). School fear persisted only in 5 subjects (8.6%) and normalized in 40 adolescents (70.2%) over time. In 6 subjects (10.3%) it crossed over to truancy and in another 6 subjects it went over into a combination of school refusal and truancy.

**Figure 2:**
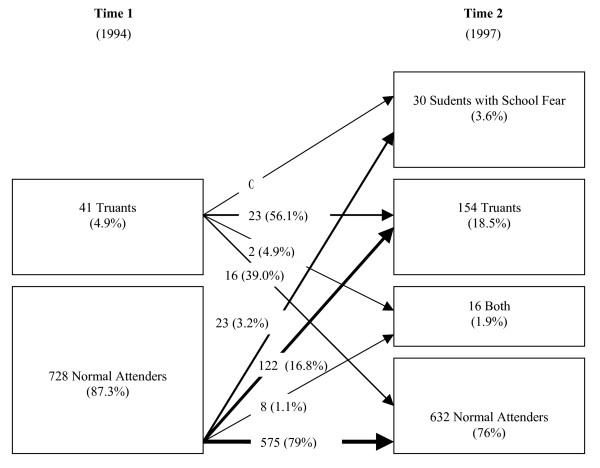
Stability of truancy from time 1 to time 2.

For truancy the frequency was 4.9% at time 1 and 18.5% at time 2. The stability of truancy is shown in figure 2. Truancy increased significantly from time 1 to time 2 (McNemar p < .001). Females were more frequently showing this behaviour at time 2 (Chi^2 ^= 7.0, p < .01). There was persistent truancy in 23 subjects (56.1%), episodic truancy in 16 adolescents (39%), and no switch over from truancy to school refusal whereas two former subjects with truancy developed both behaviours over time. A comparison of the rate of persistence shows that it is low in school refusal whereas it is high in truancy.

### Group comparisons

Scores for behavioural and emotional problems based on the YSR at time 1 are shown in Table 2. The three groups were significantly different on all eight primary scales except somatic complaints, the two secondary scales for internalizing and externalizing problems, and the total score. The group with school fear scored higher than the two other groups on the scales measuring social withdrawal, anxious/depressed, social problems, and internalizing problems. Furthermore, this group had higher scores than controls on the scales measuring thought problems, attention problems, and total problems. In contrast, the truancy group was scoring higher than the school refusal group and the controls on delinquent behaviour and externalizing problems. Both groups displaying school absenteeism were scoring higher than controls on aggressive behaviour.

**Table 2:** YSR-Scores of three groups at time 1

	Students with School Fear (N = 57) (1)		Students with Truancy (N = 41) (2)		Controls (N = 48) (3)		F (df = 2)	p	Post hoc tests
				
	Mean	SD	Mean	SD	Mean	SD			
Withdrawn	57.0	13.9	50.5	9.0	51.0	9.9	3.91	.02	1>2,3
Somatic complaints	56.0	11.8	54.4	12.0	50.0	10.5	2.64	n.s.	
Anxious/Depressed	60.3	12.5	51.1	9.8	50.0	10.8	10.86	<.001	1>2,3
Social problems	55.1	14.3	46.0	6.3	49.3	10.7	7.10	.001	1>2,3
Thought problems	57.5	11.8	53.9	12.1	51.0	9.6	4.00	.02	1>3
Attention problems	57.0	10.4	53.9	9.6	49.7	10.1	5.44	.005	1>3
Delinquent behaviour	52.0	10.2	65.4	12.3	49.0	8.5	26.86	<.001	2>1,2
Aggressive behaviour	54.8	11.2	54.7	11.2	49.0	9.4	3.09	.05	1,2>3
Internalizing problems	59.9	12.5	52.1	8.9	50.2	10.8	8.84	<.001	1>2,3
Externalizing problems	54.3	10.8	58.9	11.4	48.9	9.1	7.38	.001	2>1>3

Total problems	59.3	11.3	54.9	9.2	49.7	9.4	8.74	<.001	1>3

Primary Scales: WILKS LAMBDA = .565, F = 5.48, df = 16; 266, p = <.001

Secondary Scales: WILKS LAMBDA = .784, F = 8.97, df = 4; 278, p = <.001

The corresponding comparisons based on the assessment at time 2 are shown in Table 3. Again, there was a significant differentiation between the three groups across all levels of the YSR. The school fear group scored higher than the two other groups on the scales measuring social withdrawal, anxious/depressed, social problems, and internalizing problems. The group with truancy scored higher than the two other groups on delinquent behaviour and externalizing problems, and higher than the controls on anxious/depressed, aggressive behaviour and internalizing problems. Both groups with school absenteeism were scoring higher than controls on somatic complaints, thought problems, and attention problems.

**Table 3:** YSR-Scores of three groups at time 2

	Students with School Fear N = 30) (1)		Students with Truancy (N = 154) (2)		Controls (N = 90) (3)		F (df = 2)	p	Post hoc tests
				
	Mean	SD	Mean	SD	Mean	SD			
Withdrawn	56.5	12.6	51.4	10.0	49.6	8.2	3.67	.03	1>2,3
Somatic complaints	56.9	12.9	53.6	10.6	49.1	9.5	5.11	.007	1>3;2>3
Anxious/Depressed	56.6	13.0	52.6	10.6	48.3	8.4	9.60	<.001	1>2,3; 2>3
Social problems	58.0	12.1	48.9	8.5	48.4	7.8	14.18	<.001	1>2,3
Thought problems	55.2	12.7	53.5	11.6	48.3	7.5	7.96	<.001	1>3;2>3
Attention problems	54.1	13.7	54.8	10.3	47.9	9.0	14.11	<.001	1>3;2>3
Delinquent behaviour	50.4	9.5	60.5	10.8	46.7	6.6	59.03	<.001	2>1,3
Aggressive behaviour	51.5	10.2	55.1	10.8	48.4	9.4	12.93	<.001	2>3
Internalizing problems	59.6	13.5	52.9	9.9	48.6	8.5	9.13	<.001	1>2,3;2>3
Externalizing problems	51.3	10.2	57.9	10.5	47.5	8.0	32.73	<.001	2>1,3

Total problems	57.5	13.4	55.5	9.9	47.7	8.1	19.98	.000	1,2>3

Primary Scales: WILKS LAMBDA = .578, f = 5.48, F = 10.29, df = 16; 522, p = <.001

Secondary Scales: WILKS LAMBDA = .768, f = 8.97, F = 18.84, df = 4; 534, p = <.001

Further comparisons of the groups dealt with a number of other psychosocial variables. Findings at time 1 are given in Table 4 and show that there were significant differences between the three groups with regard to self-esteem, performance stress, possibility to participate and peer acceptance at school. Subjects with school fear showed less self-esteem than the two other groups, experienced more performance stress and less possibility to participate than controls, and felt less accepted by their peers than truants.

**Table 4:** Psychosocial Variables of three groups at time 1

	Students with School Fear (N = 57) (1)		Students with Truancy (N = 41) (2)		Controls (N = 48) (3)		F (df = 2)	p	Post hoc tests
				
	Mean	SD	Mean	SD	Mean	SD			
Life events	56.2	11.8	54.2	10.4	53.0	8.8	1.06	n.s.	
Life events impact	42.9	13.4	47.6	10.3	47.4	9.2	2.43	n.s.	
Self-esteem	42.9	11.5	49.0	11.0	51.4	8.7	9.11	<.001	1<2,3
Self-awareness	53.4	10.9	50.2	7.2	50.1	9.3	1.13	n.s.	
Maternal acceptance	47.9	11.2	47.7	10.3	50.3	10.9	.43	n.s.	
Maternal rejection	53.9	11.8	51.8	11.9	49.3	9.1	.92	n.s.	
Maternal control	49.8	9.7	47.4	11.4	49.2	9.4	.98	n.s.	
Paternal acceptance	48.4	11.8	48.0	10.7	48.5	12.7	.08	n.s.	
Paternal rejection	52.1	10.8	50.8	10.6	50.1	11.2	.16	n.s.	
Paternal control	48.4	10.8	47.6	10.4	49.7	8.4	.97	n.s	
Competition among pupils	55.9	11.6	51.3	9.8	52.2	9.8	3.12	.05	
Control by the teacher	54.8	12.3	53.7	9.0	50.3	9.2	2.10	n.s.	
Performance stress	55.4	11.2	52.5	8.2	49.9	10.2	4.38	.01	1>3
Possibility to participate	45.3	10.7	47.0	9.4	50.1	10.8	3.02	.05	1<3
Peer acceptance	44.5	11.6	52.4	7.3	49.5	11.2	7.53	.001	1<2

	WILKS LAMBDA	F	df	p					
					
Life events	.957	1.49	4; 272	n.s.					
Self-related cognitions	.878	4.67	4; 276	.001					
Maternal behaviour	.968	.76	6; 276	n.s.					
Paternal behaviour	.977	.53	6; 274	n.s.					
School environment	.825	2.73	10; 270	.003					

A final analogous comparison was made with the data based on time 2 assessments and findings are shown in Table 5. The two life event-scores differentiated significantly between the three groups. At this time, both groups with different forms of school absenteeism scored higher with regard to total number of life events and experienced more negative impact than the controls. There were significant differences between the groups with regard to self-related cognitions. The group with school fear showed less self-esteem than the other two groups and both groups with school absenteeism had more self awareness than controls.

**Table 5:** Psychosocial Variables of three groups at time 2

	Students with School Fear (N = 30) (1)		Students with Truancy (N = 154) (2)		Controls (N = 90) (3)		F (df = 2)	p	Post hoc tests
				
	Mean	SD	Mean	SD	Mean	SD			
Life events	54.3	11.5	55.2	10.2	48.7	8.7	12.82	<.001	1,2>3
Life events impact	44.6	11.9	46.4	10.7	50.9	9.2	6.45	.002	3>1,2
Self-esteem	41.9	10.8	47.8	9.8	51.0	9.1	8.59	<.001	1<2,3
Self-awareness	54.7	9.1	53.5	9.1	49.1	9.5	10.33	<.001	1,2>3
Maternal acceptance	52.2	8.9	48.5	10.4	52.5	8.6	5.24	.006	2<3
Maternal rejection	53.3	9.5	52.4	11.1	48.2	8.3	6.42	.002	2>3
Maternal control	52.0	7.9	47.6	11.0	50.0	9.1	3.42	.03	
Paternal acceptance	50.4	10.7	47.6	10.7	52.5	8.8	6.27	.002	2<3
Paternal rejection	56.4	12.2	53.1	11.1	48.7	8.5	8.97	<.001	1,2>3
Paternal control	52.5	8.3	48.4	10.9	51.6	9.3	4.03	.02	
Competition among pupils	59.1	11.1	51.6	9.9	48.1	9.4	3.91	.02	1>2,3
Control by the teacher	54.8	10.5	52.2	9.9	47.6	8.7	7.63	.001	1,2>3
Performance stress	55.3	10.3	51.7	9.2	49.3	9.4	.97	n.s.	
Possibility to participate	48.2	9.2	46.0	10.4	52.1	9.1	.62	n.s.	
Peer acceptance	42.6	10.9	51.9	9.0	51.2	9.2	.29	n.s.	

	WILKS LAMBDA	F	df	p					
					
Life events	.898	7.34	4; 532	<.001					
Self-related cognitions	.896	7.40	4; 524	<.001					
Maternal behaviour	.883	5.61	6; 526	<.001					
Paternal behaviour	.841	7.36	6; 488	<.001					

Subjects with truancy felt less accepted by both parents and more rejected by the mother than controls and both school absenteeism groups felt more rejected by the father than controls. Finally, there were also significant differences between the groups when the school environment scales were compared. Perceived competition among pupils was higher among the group with school fear than in the two other groups. Teacher control was experienced significantly higher among both groups with school absenteeism than controls.

## Discussion

In the present study, the identification of subjects with school fear and truancy was based on a large community sample of adolescents who took part in a longitudinal survey. Certainly, the origin and the size of the samples are an advantage over many previous studies that had been based on rather small and selected sample. However, comparisons to the literature are also hampered by the different design of the present study.

The two subsamples were defined only by two self-reported proxy items rather than a full clinical assessment or school reports on real absences from school. Whereas self-reports by the adolescents themselves have to be considered a more reliable information rather than parental reports, the present study does not contain information on the extension, duration, and motivation of school-absenteeism in the subjects of the various subsamples because these data had not been collected in the original survey. Furthermore, the YSR index of truancy probably is better than the YSR indicator of school fear. These restrictions of the present study have to be born in mind for the following discussion of the findings.

With frequencies for school fear of 6.9% at a mean age of thirteen years and 3.6% at a mean age of sixteen years, the present study is reporting figures that are not significantly deviating from other reports in the literature with frequency rates between 1 and 5 per cent [1,2]. The declining trend over a mean interval of three years is also matching findings in these previous studies. Similarly, both the frequencies for truancy of 4.9% at a mean age of thirteen years and of 18.5% at a mean age of sixteen years and the increasing trend with age are consistent with previous findings [3]. The high rate of stability of truancy is also matching these findings.

There is some controversy in the literature whether or not there are significant gender differences in the manifestation of school refusal and truancy with some studies denying any differences [1,2], whereas others are pointing to trends for females showing school refusal and males showing truancy more frequently [7,15]. In the present study, there was a significantly higher rate of females in the school fear group at both times and in the truancy group at time 2. To some extent, this finding may be influenced by a reporting bias with females perhaps being more willing to confess both forms of school absenteeism. On the other hand, the present findings contribute to observations of marked recent societal changes in adolescent behaviour with females behaving very similar to males or even exceeding the rates of abnormal behaviour of males when it comes to behaviours like smoking, self-mutilation, or even conduct problems including truancy.

The group comparisons at time 1 and time 2 across the various domains of emotional and behavioural problems were largely in accordance with what could be expected from clinical findings. Findings from both times of assessment show that school fear is predominantly associated with a pattern of various internalizing problems and additional social problems, whereas there is a strong link between truancy and delinquent behaviour as well as externalizing problems. At a younger age around a mean of thirteen years school fear showed also an association with aggressive behaviour that was different from controls and later at a mean age of sixteen years truancy was amalgamated with some features of internalizing problems that again were different from controls.

Taken together, these findings based on a community study are very much in accordance with clinical findings pointing to a predominant association of school refusal with internalizing problems [6-8], and truancy with externalizing problems [10,11] in children and adolescents. However, it should be noted that the mean YSR scores of the subsamples of the present study are not in the clinical range of functioning emphasizing the differences between community and clinical samples.

Further differentiation of school fear and truancy came from the comparison of a large group of other psychosocial variables. However, the pattern of differentiation at the two times of assessment was different. There was no contribution of life events at time 1. However, at time 2 in both groups of school absentees life event variables were significantly increased both in terms of frequency and negative impact. Thus, with a decrease of the numbers of subjects with school fear and an increase of the numbers of truants across time during adolescence the association with life events became stronger. Given the very limited knowledge about the contribution of life events to school absenteeism, the present finding is noteworthy. It converges with the finding by Huffington and Sevitt [13] indicating a significant though not specific increase of life events in these subjects. One may speculate that with increasing age adolescents showing some form of school absenteeism become more sensitive to life events and their impact or are at least more reliably reporting their life events. The general increase of life events in maladjusted children and adolescents is a well-known fact and has been found also in the ZAPPS both as a general risk factor [5] and as a specific risk factor for various groups of subjects suffering from depression [32], suicidal ideation [33,34], eating disorders [35], and substance abuse [36,37].

The two personality variables reflecting self-related cognitions showed that subjects with school fear had less self-esteem than the other two groups at both times and both students with school fear and truants displayed increased amounts of self-awareness at both times when compared with controls. These findings correspond well to the pattern of internalizing problems of school refusers and reflect the component of critical introspection and depressive features that is inherent to the syndrome of school refusal in many clinical cases. The increase in self-awareness in students with school fear matches these features. The fact that truancy was also associated with higher scores in self-awareness in older adolescents with a larger proportion of females at time 2 should guard against stereotyping these subjects as being neither reflective nor introspective.

The perceived parenting behaviour did not contribute at all to the differentiation of the younger adolescents at time 1 and more to the differentiation of truants than of subjects with school fear from controls later in adolescence at time 2. The former felt less accepted and more rejected by both parents, whereas students with school fear experienced only more paternal rejection than controls. Thus, in the present study an association between school absenteeism and perceived parental behaviour became obvious only later in time at the height of adolescence and predominantly in truants. This perceived deficit of the parents may well be a correlate of the actual neglecting and rejecting parent behaviour in truants that has been described by others [15,21]. A criticizing father has also been described as being significant for school refusers [13].

Finally, the perceived school environment allowed some important differentiation of the students with school fear at the two times of the study. Although the pattern of distress was not identical at the two times, they were the group who suffered most from the school environment. They were suffering from more distress than controls due to increased performance stress and lacking possibility to participate and in comparison to truants from a lack of peer acceptance at time 1. Later in adolescence at time 2, these subjects had greater problems with competition at school than the two other groups and together with truants felt more controlled by the teacher than the controls. Thus, these differentiating characteristics do not only fit well into the clinical picture of school refusal with perceived distress coming from the school environment as described by others [22,23] but also serve as a validation of the group definition that has been taken in the present study.

Limitations of the present study are due to the restricted definition of school absenteeism with no actual figures of the actual amount of school refusal and truancy. These figures could have been obtained only by information coming from the schools because parents often are ill-informed about school absenteeism of their youngsters. However, collecting data about actual school absenteeism was not a part of the original study design. Thus, information by the adolescents was considered to provide the best evidence that was available.

## Conclusion

Despite a very simple definition by two items of a self-reported checklist for children and adolescents the present study did not only allow the assessment of the frequency and stability of self-reported school fear and truancy but also a clinically meaningful differentiation of these two forms of school absenteeism by further behavioural and psychosocial characteristics. Furthermore, various insights derived from selected clinical samples were corroborated by this unselected community study. Finally, the large number of non existing differences between the school refusal and truancy groups may not only reflect the above mentioned restrictions due to measurement by two proxy items only but may, rather, be also a consequence of overlapping characteristics of the two entities.

## Competing interests

The authors declare that they have no competing interests.

## Authors' contributions

HCS designed the study and drafted the manuscript. NM and CWM performed the statistical analyses. CWM participated in the design and coordination of the study. All authors read and approved the final manuscript.

## References

[B1] King NJ, Bernstein GA (2001). School refusal in children and adolescents: a review of the past 10 years. J Am Acad Child Adolesc Psychiatry.

[B2] Fremont WP (2003). School refusal in children and adolescents. Am Fam Physician.

[B3] Kearny CA (2008). School absenteeism and school refusal behavior in youth: A contemporary review. Clin Psychology Rev.

[B4] Henry KL, Huizinga DH (2007). Who's skipping school: characteristics of truants in 8^th ^and 10^th ^grade. J School Health.

[B5] Steinhausen H-C, Winkler Metzke C (2001). Risk, compensatory, vulnerability, and protective factors influencing mental health in adolescence. J Youth Adolesc.

[B6] Bernstein GA (1991). Comorbidity and severity of anxiety and depressive disorders in a clinic sample. J Am Acad Child Adolesc Psychiatry.

[B7] Berg I, Last CG (1993). Aspects of school phobia. Anxiety across the lifespan: a developmental perspective.

[B8] Kearney CA (2001). School refusal behavior in youth.

[B9] Egger HL, Costello EJ, Angold A (2003). School refusal and psychiatric disorders: a community study. J Am Acad Child Adolesc Psychiatry.

[B10] Miller P, Plant M (1999). Truancy and perceived school performance: an alcohol and drug study of UK teenagers. Alcohol Alcohol.

[B11] Pritchard C, Cotton A, Cox M (1992). Truancy and illegal drug use, and knowledge of HIV infection in 932 14–16-year-old adolescents. J Adolesc.

[B12] Henry KL (2007). School-related risk and protective factors associated with truancy among urban youth placed at risk. J Prim Prev.

[B13] Huffington CM, Sevitt MA (1989). Familiy interaction in adolescent school phobia. J Fam Thera.

[B14] Southworth P (1992). Psychological and social characteristics associated with persistent absence among secondary aged school children with special reference to different categories of persistent absence. Personality and Individual Differences.

[B15] Reid K (1984). Some social, psychological and educational aspects to persistent school absenteeism. Res Edu.

[B16] Bernstein GA, Svingen PH, Garfinkel BD (1990). School phobia: patterns of family functioning. J Am Acad Child Adolesc Psychiatry.

[B17] Bernstein G, Borchardt C (1996). School refusal: family constellation and family functioning. J Anx Dis.

[B18] Corville-Smith J, Ryan BA, Adams GR, Dalicandro T (1998). Distinguishing absentee students from regular attenders: the combined influence of personal, family, and school factors. J Youth Adolesc.

[B19] Bernstein GA, Garfinkel BD (1988). Pedigrees, functioning, and psychopathology in families of school phobic children. Am J Psychiatry.

[B20] Galloway D, Hersov L, Berg I (1980). Problems in the assesssment and management of persistent absenteeim from school. Out of school.

[B21] Farrington D, Hersov L, Berg I (1980). Truancy, delinquency, the home and the school. Out of school.

[B22] Moos RH, Moos BS (1978). Classroom social climate and student absences and grades. J Edu Psychology.

[B23] Reynolds D, Jones D, St Leger S, Murgatroyd S, Hersov L, Berg I (1980). School factors and truancy. Out of school.

[B24] Steinhausen H-C, Metzke CW, Meier M, Kannenberg R (1998). Prevalence of child and adolescent psychiatric disorders: the Zurich epidemiological study. Acta Psychiatr Scand.

[B25] Achenbach TM (1991). Manual for the youth self-report and 1991 profile.

[B26] Steinhausen H-C, Winkler Metzke C (1998). Youth self report of behavioral and emotional problems in a Swiss epidemiological study. J Youth Adolesc.

[B27] Steinhausen H-C, Winkler Metzke C (2001). Die Zürcher Lebensereignis-Liste (ZLEL): Ergebnisse einer epidemiologischen Untersuchung. [The Zurich life event list (ZLEL): Findings from an epidemiological study]. Kindheit und Entwicklung.

[B28] Rosenberg M (1965). Society and the adolescent self-image.

[B29] Filipp S-H, Freudenberg E (1989). Der Fragebogen zur Erfassung dispositionaler Selbstaufmerksamkeit [Questionnaire for the assessment of dispositional self – awareness].

[B30] Reitzle M, Winkler Metzke C, Steinhausen H-C (2001). Eltern und Kinder: Der Zürcher Kurzfragebogen zum Erziehungsverhalten (ZKE). [Parents and children: The Zurich Short Questionnaire on parental rearing behavour (ZKE)]. Diagnostica.

[B31] Fend H, Prester H-G (1986). Bericht aus dem Projekt "Entwicklung im Jugendalter" [Report from the project 'development in adolescence']. Bericht aus dem Projekt "Entwicklung im Jugendalter" [Report from the project 'Development in adolescence'].

[B32] Steinhausen H-C, Winkler Metzke C (2001). Adolescent self-rated depressive symptoms in a Swiss epidemiological study. J Youth Adoles.

[B33] Steinhausen H-C, Boesiger R, Winkler Metzke C (2006). Stability, correlates, and outcome of adolescent suicidal ideation. J Child Psychol Psychiatriy.

[B34] Steinhausen HC, Winkler Metzke CW (2004). The impact of suicidal ideation in preadolescence, adolescence, and young adulthood on psychosocial functioning and psychopathology in young adulthood. Acta Psychiatr Scand.

[B35] Steinhausen HC, Gavez S, Winkler Metzke C (2005). Psychosocial correlates, outcome, and stability of abnormal adolescent eating behavior in community samples of young people. Int J Eat Disord.

[B36] Steinhausen HC, Winkler Metzke C (1998). Frequency and correlates of substance use among preadolescents and adolescents in a Swiss epidemiological study. J Child Psychol Psychiatry.

[B37] Steinhausen HC, Metzke CW (2003). The validity of adolescent types of alcohol use. J Child Psychol Psychiatry.

